# Mortality in the Russian Capitols

**Published:** 1860-07

**Authors:** 


*Mortality in the Russian Capitals.*—At St. Petersburg, the number of births in 1828 amounted to 17,658 (9147 boys, and 8511 girls), while the deaths were 19,077. This fact, which occurs every year, demonstrates that, as the deaths exceed the births, the population of that city is only kept up by immigration. The number of illegitimate births increases, while marriages are diminishing. At Moscow, the number of births was 11,267 (5822 boys, and 5445 girls), and that of deaths, 11,703.

